# Structure-based mechanism of cysteine-switch latency and of catalysis by pappalysin-family metallopeptidases

**DOI:** 10.1107/S2052252519013848

**Published:** 2020-01-01

**Authors:** Tibisay Guevara, Arturo Rodriguez-Banqueri, Miroslaw Ksiazek, Jan Potempa, F. Xavier Gomis-Rüth

**Affiliations:** a Proteolysis Laboratory, Department of Structural Biology, Molecular Biology Institute of Barcelona, CSIC, Barcelona Science Park, Helix Building, c/ Baldiri Reixac, 15-21, 08028 Barcelona, Catalonia, Spain; b Department of Oral Immunology and Infectious Diseases, University of Louisville School of Dentistry, 501 South Preston Street, Louisville, KY 40202, USA; c Department of Microbiology, Faculty of Biochemistry, Biophysics and Biotechnology, Jagiellonian University, ul. Gronostajowa 7, Kraków 30-387, Poland

**Keywords:** pappalysin family, metallo­peptidases, mirolysin, peridontopathogens, zymogens, catalytic mechanisms

## Abstract

*Tannerella forsythia* is an oral dysbiotic periodontopathogen involved in severe human periodontal disease. As part of its virulence factor armamentarium, at the site of colonization it secretes mirolysin, a metallopeptidase of the unicellular pappalysin family, as a zymogen that is proteolytically auto-activated extracellularly at the Ser54–Arg55 bond. Crystal structures of the catalytically impaired promirolysin point mutant E225A at 1.4 and 1.6 Å revealed that latency is exerted by an N-terminal 34-residue pro-segment that shields the front surface of the 274-residue catalytic domain, thus preventing substrate access. The catalytic domain conforms to the metzincin clan of metallopeptidases and contains a double calcium site, which acts as a calcium switch for activity. The pro-segment traverses the active-site cleft in the opposite direction to the substrate, which precludes its cleavage. It is anchored to the mature enzyme through residue Arg21, which intrudes into the specificity pocket in cleft sub-site S_1_′. Moreover, residue Cys23 within a conserved cysteine–glycine motif blocks the catalytic zinc ion by a cysteine-switch mechanism, first described for mammalian matrix metallopeptidases. In addition, a 1.5 Å structure was obtained for a complex of mature mirolysin and a tetradecapeptide, which filled the cleft from sub-site S_1_′ to S_6_′. A citrate molecule in S_1_ completed a product-complex mimic that unveiled the mechanism of substrate binding and cleavage by mirolysin, the catalytic domain of which was already preformed in the zymogen. These results, including a preference for cleavage before basic residues, are likely to be valid for other unicellular pappalysins derived from archaea, bacteria, cyanobacteria, algae and fungi, including archetypal ulilysin from *Methanosarcina acetivorans*. They may further apply, at least in part, to the multi-domain orthologues of higher organisms.

## Abstract 2

The members of the unicellular pappalysin family of metallopeptidases are secreted as zymogens with a short N-terminal pro-segment, which blocks the catalytic zinc cation by a cysteine-switch mechanism, as shown structurally for bacterial mirolysin. This is a mechanism to prevent activity in the absence of the adequate temporal and spatial requisites. In addition, the complex of mirolysin with a large peptide reveals the structural basis of its catalytic mechanism.

## 1. Introduction   

*Tannerella forsythia* is a Gram-negative bacterium, which was first isolated from patients by Anne Tanner at The Forsyth Institute in the mid-1970s (Tanner *et al.*, 1979 ▸) and later named after her (Tanner & Izard, 2006 ▸; Tindall *et al.*, 2008 ▸). It is a member of the dysbiotic oral microbiome responsible for severe periodontal disease (PD), which is the sixth most prevalent disabling health condition that affects an estimated 750 million people worldwide (Kassebaum *et al.*, 2014 ▸; Hajishengallis, 2015 ▸). PD is a polymicrobial synergistic inflammatory disease in which a major role is exerted by the red complex, a bacterial consortium of *T. forsythia*, *Porphyro­monas gingivalis* and *Treponema denticola* that colonizes the gingival crevice and forms dental plaque biofilms (Socransky *et al.*, 1998 ▸; Holt & Ebersole, 2005 ▸). *T. forsythia* is strongly associated with destructive inflammatory host responses (Hajishengallis, 2014 ▸; Lamont & Hajishengallis, 2015 ▸). Outside the oral cavity it is linked to accelerated progression of atherosclerotic lesions in mice and an increased risk of esophageal adenocarcinoma in humans (Peters *et al.*, 2017 ▸). In addition, it has been associated with skin abscesses in animal models (Takemoto *et al.*, 1997 ▸; Bird *et al.*, 2001 ▸) and has been isolated from women with bacterial vaginosis (Cassini *et al.*, 2013 ▸). These findings underpin the capacity of the bacterium to colonize niches distal from the gingival crevice, with systemic implications probably similar to those for *P. gingivalis* (Seymour *et al.*, 2007 ▸; Olsen & Yilmaz, 2019 ▸).

Severe PD is associated with tissue destruction caused by a self-damaging inflammatory host response to the colonizing bacteria, as well as by secreted bacterial virulence factors. Among these, peptidases degrade proteins to dismantle host structures and to nourish bacteria (Dubin *et al.*, 2013 ▸). Studies of the components of *T. forsythia* outer-membrane vesicles and of the outer-membrane proteome have identified at least 13 (Friedrich *et al.*, 2015 ▸) and seven (Veith *et al.*, 2009 ▸) peptidase candidates, respectively. In addition, the cysteine peptidase PrtH (Saito *et al.*, 1997 ▸), the serine peptidase ‘trypsin-like’ (Grenier, 1995 ▸) and a cluster of peptidases belonging to different chemical classes collectively dubbed KLIKK peptidases (Ksiazek, Mizgalska *et al.*, 2015 ▸) have been described or identified in *T. forsythia*. These include the subtilisin-type serine peptidase mirolase (Ksiazek, Karim *et al.*, 2015 ▸), the trypsin-type serine peptidases miropsin-1 and -2 (Ksiazek, Mizgalska *et al.*, 2015 ▸; Eckert *et al.*, 2018 ▸), and forsilysin from the thermolysin family of the gluzincin clan of metallopeptidases (MPs) (Hooper, 1994 ▸; Cerdà-Costa & Gomis-Rüth, 2014 ▸; Ksiazek, Mizgalska *et al.*, 2015 ▸). Another KLIKK MP is karilysin (Karim *et al.*, 2010 ▸; Koziel *et al.*, 2010 ▸; Cerdà-Costa *et al.*, 2011 ▸; Jusko *et al.*, 2012 ▸; Skottrup *et al.*, 2012 ▸, 2019 ▸; Guevara *et al.*, 2013 ▸; Potempa *et al.*, 2013 ▸; López-Pelegrín *et al.*, 2015 ▸), which belongs to the matrix metalloproteinase (MMP) family within the metzincin clan. Metzincins are zinc-dependent MPs that share several structural features including a methionine-containing Met-turn and an active-site helix with an extended zinc-binding motif (HE*XX*H*XX*G*XX*H/D). The two terminal amino acids and the central histidine act as zinc ligands with glutamate as the general base/acid for catalysis (Bode *et al.*, 1993 ▸; Stöcker *et al.*, 1995 ▸; Gomis-Rüth, 2003 ▸, 2009 ▸; Cerdà-Costa & Gomis-Rüth, 2014 ▸). Owing to their destructive potential, metzincins and other MPs must be tightly controlled. One mechanism is biosynthesis as an inactive or latent zymogen with a blocking pro-domain or pro-segment (PS) that is removed during activation (Khan & James, 1998 ▸; Bryan, 2002 ▸; Lazure, 2002 ▸; Arolas *et al.*, 2018 ▸).

**Figure 1:**
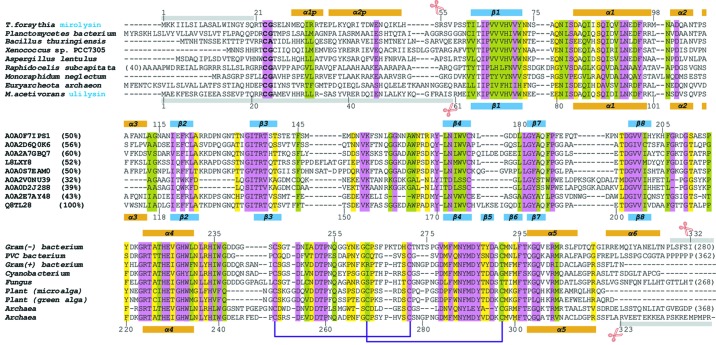
Unicellular pappalysin family members. Structure-assisted sequence alignment of selected pappalysins from prokaryotes and lower eukaryotes depicting the respective (potential) CDs and upstream PSs. The organism, the UniProt code plus the sequence identity with ulilysin in parentheses, and the organism category are displayed at the beginning of each sequence block, respectively. Very high, high and middle sequence similarities are characterized by magenta, green and yellow backgrounds, respectively. Regular secondary-structure elements (helices and strands as orange and blue bars, respectively) below and above the alignment correspond to ulilysin and (pro)mirolysin, respectively. Their numbering is consistent with that of ulilysin, see Tallant *et al.* (2006 ▸). The conserved CG-motif responsible for latency in promirolysin is shown in bold. The number of additional N- and C-terminal residues is shown in parentheses. Residues not present in the structure of native promirolysin (this work; PDB entry 6r7v) and mature ulilysin (PDB entry 2cki) are denoted by grey bars above and below the alignment, respectively. The disulfides found in both ulilysin and mirolysin are shown as purple handles. Red scissors indicate autolytic activation points (P_1_′ residues) of ulilysin (Tallant *et al.*, 2006 ▸) and mirolysin (Koneru *et al.*, 2017 ▸).

The pappalysins (Boldt *et al.*, 2001 ▸; Gomis-Rüth, 2003 ▸; Tallant *et al.*, 2006 ▸; Cerdà-Costa & Gomis-Rüth, 2014 ▸; Conover & Oxvig, 2018 ▸) are another metzincin family which includes archetypal pregnancy-associated plasma protein-A (PAPP-A), which was originally identified as a human pregnancy antigen (Gall & Halbert, 1972 ▸; Lin *et al.*, 1974 ▸). PAPP-A is a glycosylated multi-domain 180 kDa protein, which contains an ∼300-residue catalytic domain (CD) that specifically hydrolyses insulin-like growth factor binding protein 4 (Lawrence *et al.*, 1999 ▸; Conover & Oxvig, 2018 ▸). Other family members are the paralogue PAPP-A2 and potential orthologues from other mammals, birds, reptiles, amphibians, fish, mollusks, nematodes and cnidarians. These sequences grossly share the length and multi-domain structure of PAPP-A. In addition, shorter sequences encompassing a CD with sequence identities of 25–30% with the archetype are present in archaea, bacteria, cyanobacteria, fungi and algae. These are hereafter referred to as the unicellular pappalysins [see Fig. 1 ▸ and Fig. 1 in the work by Tallant *et al.* (2006 ▸)]. One such pappalysin is archaeal ulilysin (alias lysargiNase) from *Methanosarcina acetivorans*, which is the only family member analysed for its three-dimensional structure and function to date (Tallant *et al.*, 2006 ▸, 2007 ▸; García-Castellanos *et al.*, 2007 ▸; Huesgen *et al.*, 2015 ▸). Another unicellular pappalysin is the *T. forsythia* KLIKK MP mirolysin, which protects the bacterium against complement-mediated bactericidal activity (Ksiazek, Mizgalska *et al.*, 2015 ▸). It is secreted as a 66 kDa zymogen (see UniProt code A0A0F7IPS1), which when activated cleaves several physiologically relevant host proteins such as fibronectin; fibrinogen; complement proteins C3, C4 and C5; as well as antimicrobial peptide LL-37 (Koneru *et al.*, 2017 ▸).

Here, we determine the mechanisms of latency and catalysis of unicellular pappalysins by high-resolution crystal structure analysis of the mirolysin zymogen and a product complex.

## 2. Materials and methods   

### 2.1. Protein production and purification   

The coding sequence of *T. forsythia* strain ATCC 43037 promirolysin, without the signal peptide and with or without the E225A mutation, was cloned into a vector for overexpression in *Escherichia coli* BL21 (DE3) cells as a fusion construct with N-terminal glutathione S-transferase and a PreScission endopeptidase target sequence as previously described (Koneru *et al.*, 2017 ▸). The recombinant promirolysin variants comprised residues Gln20–Ser331 preceded by a glycine–proline dipeptide, because of the cloning strategy, and were purified by glutathione Sepharose affinity and size-exclusion chromatographies. A variant of the protein in which methionine was replaced with selenomethionine was obtained in the same way except that the modified amino acid was used instead of the natural residue in minimal cell culture medium. Recombinant mature wild-type mirolysin was prepared as previously described (Koneru *et al.*, 2017 ▸) and spanned residues Arg55–Ser331. The protein was incubated at a 1:2 molar ratio with the small lipoprotein BFO_2662 (UniProt code G8ULV2) during crystallization studies (see Section 2.2), which cleaved the protein. Its C-terminal segment remained bound to mirolysin in a product complex.

### 2.2. Crystallization and diffraction data collection   

Proteins were crystallized by the sitting-drop vapour diffusion method. Reservoir solutions were mixed in plates of 96 × 2 ml deep wells with a Tecan robot. A Phoenix robot (Art Robbins) dispensed nanodrops of protein and reservoir solutions into MRC plates of 96 × 2 ml wells (Innovadyne). Several hundreds of conditions from multiple screenings were assayed at the joint IBMB/IRB Automated Crystallography Platform of Barcelona Science Park. Plates were stored at 4 or 20°C in Bruker steady-temperature crystal farms. The best native and selenomethionine-derivatized promirolysin crystals were obtained at 20°C from drops with a 200 nl protein solution at ∼0.6 mg ml^−1^ in 5 m*M* Tris HCl, 50 m*M* sodium chloride, pH 8.0 and a 100 nl reservoir solution, comprising 25% polyethylene glycol (PEG) 1500, 0.1 *M* MIB buffer (malonic acid, imidazole and boric acid at a 2:3:3 molar ratio) at pH 6.0. The best crystals of the mirolysin product complex were obtained with protein solution at ∼12 mg ml^−1^ in 5 m*M* Tris HCl, 50 m*M* sodium chloride, 5 m*M* calcium chloride and pH 8.0 at 4°C from drops containing a 200 nl protein solution, and 100 nl of a reservoir solution of 40% ethanol, 5% PEG 1000, and 0.1 *M* phosphate–citrate buffer at pH 4.2.

Crystals were cryo-protected by rapid passage through drops containing reservoir solution plus 10–15% glycerol(*v*/*v*) and flash vitrified in liquid nitrogen before transport to the ALBA synchrotron in Cerdanyola (Catalonia, Spain). Diffraction data were collected at the zinc absorption edge from cryo-cooled crystals on a PILATUS 6M pixel detector (Dectris) at beamline XALOC (Juanhuix *et al.*, 2014 ▸). The data were indexed, integrated and merged by programs *XDS* (Kabsch, 2010*a* ▸) and *XSCALE* (Kabsch, 2010*b* ▸). Data were transformed with *XDSCONV* to MTZ format for structure solution and refinement. The native promirolysin crystals belonged to space group *P*2_1_2_1_2_1_, contained one molecule per asymmetric unit and were processed to 1.4 Å resolution. The selenomethionine-containing promirolysin crystals belonged to space group *P*2_1_, had two protein molecules (A and B) per asymmetric unit and were processed to 1.6 Å resolution. Finally, the crystals of mirolysin in a product complex belonged to space group *P*2_1_2_1_2_1_, contained one complex per asymmetric unit and were processed to 1.5 Å resolution. Table 1 ▸ provides a summary of the data-processing statistics.

### 2.3. Structure solution and refinement   

The structure of selenomethionine-derivatized promirolysin was solved first by a combination of single-wavelength anomalous diffraction with the *Autosol* routine (Terwilliger *et al.*, 2009 ▸) of the *PHENIX* program suite (Adams *et al.*, 2010 ▸) and maximum-likelihood-scored molecular replacement with the *Phaser* program (McCoy *et al.*, 2007 ▸). For these calculations, we used a dataset collected at the zinc absorption peak processed with separate Friedel pairs (see Table 1 ▸) and the coordinates of the protein part of *M. acetivorans* mature ulilysin [Arg61–Ala322, PDB entry 2cki, Tallant *et al.* (2006 ▸)], which had been pruned with the *CHAINSAW* program (Stein, 2008 ▸) according to a sequence alignment with mirolysin performed with *MultAlin* (Corpet, 1988 ▸). Two solutions were obtained at final Eulerian angles (α, β, γ) of 230.2, 119.0, 267.0° and 44.6, 99.5, 240.9°; with fractional cell coordinates (*x*, *y*, *z*) −0.552, −0.502, 0.416 and −0.485, 0.695, −0.100, respectively. The initial values for the rotation/translation function *Z* scores were 16.1/14.3 and 14.1/12.8, respectively, and the final log-likelihood gain was 1246. The phases derived from the correspondingly rotated and translated coordinates were then used to calculate an anomalous difference Fourier map with the *CCP*4 suite (Winn *et al.*, 2011 ▸), which revealed the position of the two catalytic zinc ions. These positions, the protein coordinates and the zinc-edge dataset were fed into *phenix.autosol*, which produced a Fourier map and a model that was completed in subsequent cycles of manual model building with the *Coot* program (Emsley *et al.*, 2010 ▸) and crystallographic refinement. The latter was carried out with *PHENIX* (Afonine *et al.*, 2012 ▸) and *BUSTER/TNT* (Smart *et al.*, 2012 ▸) against data processed with merged Friedel mates (Table 1 ▸). Calculations included translation/libration/screw-rotation refinement and, initially, non-crystallographic symmetry restraints. Anisotropic *B*-factor refinement was assayed with *PHENIX* but it did not produce better statistics and maps than those from isotropic refinement (*R* factor/free *R* factor of 15.3/19.8 *versus* 16.1/18.8, respectively), so this approach was not pursued. The incorporation of selenomethionine instead of methionine was only partial, as revealed by an occupancy refinement step with all selenium atoms grouped (75% on average). The final refined model comprised residues Arg21–Pro327 from molecule A and Arg21–Leu328 from molecule B, plus two calcium ions and one zinc ion each. Four glycerols, one boric acid and 445 solvent molecules completed the model. Residues Asn164 and Gly256 of either protein molecule were Ramachandran outliers but unambiguously resolved in the final Fourier map. Moreover, respective residue Cys23 was oxidized to *S*-oxocysteine, and segments Lys51–His53 of molecule A and Gly49–His53 of molecule B were partially flexible and traced based on weak Fourier map density. Table 1 ▸ provides statistics of the final refinement.

The structure of native promirolysin was solved by molecular replacement as above using the partially refined coordinates of molecule A of the selenomethione-derivatized structure. A clear solution was found at α, β, γ, *x*, *y*, *z* values of 238.6, 93.8, 266.1°, 0.119, 0.841 and 0.366, which had initial rotation and translation function *Z* scores of 7.2 and 8.4, respectively, and a final log-likelihood gain of 9226. Subsequently, a round of automatic density modification and tracing with *ARP/wARP* (Langer *et al.*, 2008 ▸) produced a Fourier map for completing the model as above. The final refined model comprised residues Arg21–Pro327 plus one zinc and two calcium ions, four glycerols and 274 solvent molecules. Residues Asn164, Gly256 and Asn293 were Ramachandran outliers but they were unambiguously defined in the final Fourier map. Segment Leu52–Arg55 was partially flexible and traced based on weak Fourier map density; residue Cys23 was in a reduced state. See Table 1 ▸ for final-refinement statistics.

Finally, the structure of the mirolysin product complex was solved by molecular replacement using the coordinates of the mature part of native promirolysin (Arg55–Pro327) and the two calcium ions, which provided a solution at α, β, γ, *x*, *y*, *z* values of 334.0, 156.6, 346.0°, 0.199, −0.527 and 0.147. This solution had initial rotation and translation function *Z* scores of 7.3 and 10.4, respectively, and a final log-likelihood gain of 7437. The presence of a strong peak (>19σ) at the omitted zinc site confirmed the correctness of the solution. Autotracing, model building and refinement proceeded as with native promirolysin. The final refined model comprised protein residues Pro58–Pro327 (molecule A) and peptide residues *Lys1*–*Lys14* plus a citrate (*CIT−1*) constituting molecule B (residues and numbers in italics), in addition to one zinc and two calcium ions. The citrate and the sequence of the first seven residues of the peptide could be unambiguously assigned owing to the very high resolution and quality of the Fourier map. This demonstrated that the peptide corresponded to segment K110-RDPVYFIKLSTI-K123 of protein BFO_2662. Two ethanol and 348 solvent molecules completed the model. Residue Asn164 was a Ramachandran outlier that was unambiguously resolved in the final Fourier map. Table 1 ▸ provides statistics of the final refinement. In all structures, disulfides linked Cys243 with Cys271 and Cys262 with Cys291. The peptide bonds preceding Pro215, Pro266 and Pro276 were in a *cis* conformation.

### 2.4. Bioinformatics   

Structure figures were prepared with the *Chimera* program (Pettersen *et al.*, 2004 ▸). Structure superimpositions were performed with *SSM* (Krissinel & Henrick, 2004 ▸) within *Coot*. Protein interfaces were analysed with *PISA* (https://www.ebi.ac.uk/pdbe/pisa) (Krissinel & Henrick, 2007 ▸), with the interface of a complex defined as half of the sum of the buried surface areas of either molecule. Sequence similarity searches were performed with the *PSI-BLAST* protocol at NCBI (https://blast.ncbi.nlm.nih.gov/Blast.cgi) or the *BLAST* protocol at UniProt (https://www.uniprot.org/blast) using default parameters. Sequence identities were calculated by *SIM* with default parameters (https://web.expasy.org/sim/). Signal peptides were predicted with *Phobius* (http://phobius.sbc.su.se) (Käll *et al.*, 2007 ▸) or *SignalP* v.5.0 (http://www.cbs.dtu.dk/services/SignalP-5.0) (Almagro Armenteros *et al.*, 2019 ▸). A structure-assisted alignment was performed with *T-Coffee* (http://tcoffee.crg.cat/apps/tcoffee/do:expresso) (Armougom *et al.*, 2006 ▸) and then manually adjusted. The quality of the final models was assessed with the wwPDB X-ray structure validation server (https://www.wwpdb.org/validation) (Berman *et al.*, 2003 ▸). The final coordinates of selenomethionine-derivatized and native promirolysin as well as the mature mirolysin product complex are available from the PDB (codes 6r7u, 6r7v and 6r7w, respectively).

## 3. Results and discussion   

### 3.1. Crystallization of mirolysin variants   

Recombinant promirolysin undergoes zinc- and calcium-dependent step-wise autolytic processing and activation to mature 31 kDa mirolysin through truncations at both the N- and C-terminus (Koneru *et al.*, 2017 ▸), as also reported for ulilysin (Tallant *et al.*, 2006 ▸). A variant, in which the general base/acid Glu225 was replaced with alanine (E225A), lacked activity (Koneru *et al.*, 2017 ▸). This variant was used to obtain the intact zymogen for structural studies. Previously, this strategy has proven successful for other MP zymogens (Guevara *et al.*, 2010 ▸; Goulas *et al.*, 2011 ▸; Arolas *et al.*, 2012 ▸, 2016 ▸; López-Pelegrín *et al.*, 2015 ▸). We obtained two different crystal forms for native and selenomethionine-derivatized promirolysin E225A, which contained one and two protomers per asymmetric unit and diffracted to resolutions of 1.4 and 1.6 Å, respectively (Table 1 ▸). These structures contained residues Arg21–Pro328, and superposition of the native promirolysin onto the two selenomethionine-derivatized protomers revealed close similarity and r.m.s.d. values of 0.48 and 0.42 Å for the common C^α^ atoms, so they are hereafter considered equivalent. The only significant deviation was observed for segment Lys51–Arg55, which contained the final activation cleavage point (Ser54–Arg55) (Koneru *et al.*, 2017 ▸) and was flexible. Jointly, these structures provided the molecular determinants of promirolysin latency (see Sections 3.2 and 3.3), which was compared with that of other MPs (see Section 3.4).

Mature wild-type mirolysin was incubated with small lipoprotein BFO_2662 (UniProt code G8ULV2), whose coding gene is immediately upstream of mirolysin in the genome of *T. forsythia* (Ksiazek, Mizgalska *et al.*, 2015 ▸), and the mixture was set up for crystallization. We obtained a structure at 1.5 Å resolution (Table 1 ▸) with one copy of mirolysin per asymmetric unit (Arg55–Pro327). With minor exceptions, this fourth protomer was very similar to the CD of promirolysin (see Section 3.5). However, a tetradecapeptide from the lipoprotein was found covering sub-sites S_1_′ to S_6_′ and more of the primed side of the active-site cleft [for sub-site and peptide substrate nomenclature, see Schechter & Berger (1967 ▸) and Gomis-Rüth, Botelho *et al.* (2012 ▸)]. Moreover, a citrate anion was attached to S_1_ on the non-primed side. Thus, we serendipitously trapped a product complex, which revealed the molecular determinants of substrate binding and catalysis of mirolysin (see Section 3.5).

### 3.2. The promirolysin structure   

**Figure 2:**
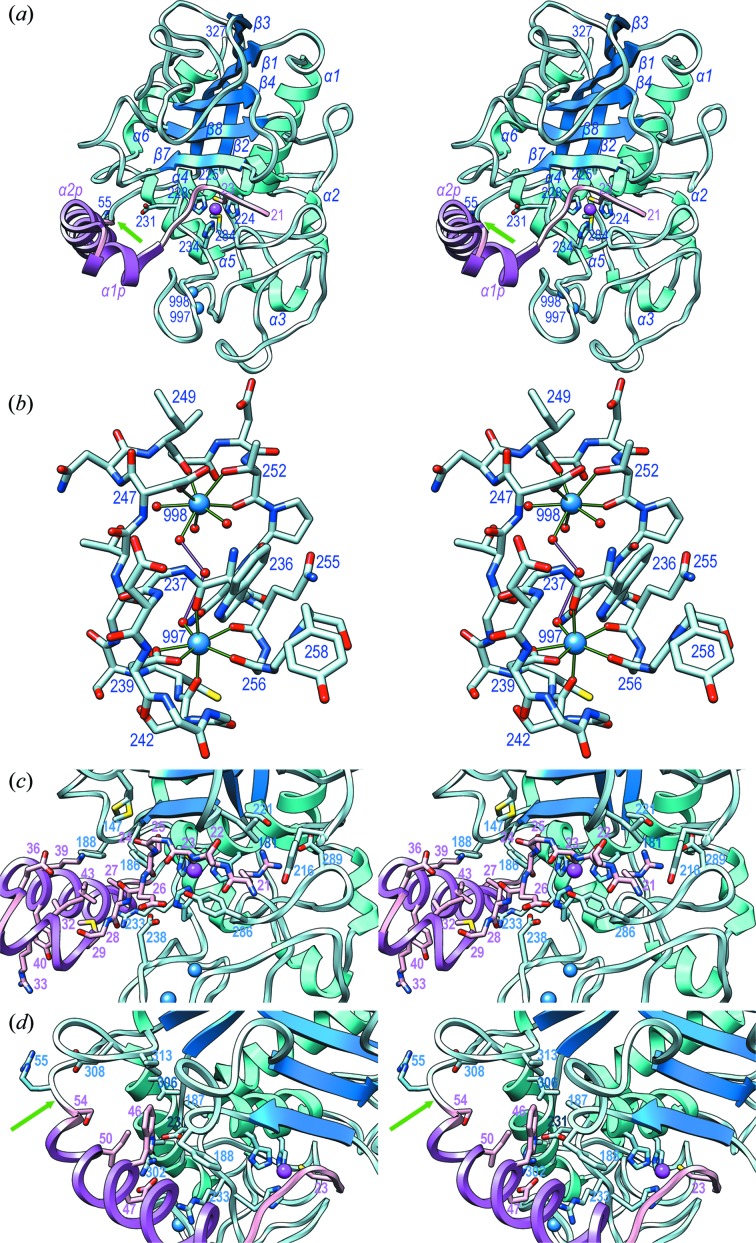
The promirolysin structure. (*a*) A ribbon-type plot in cross-eyed stereo of native promirolysin in standard orientation (Gomis-Rüth, Botelho *et al.*, 2012 ▸). The PS is in pink with the helices in magenta (α1p and α2p; for numbering, see Fig. 1 ▸). The CD is in pale blue, with the helices (α1–α6) in cyan and the β-strands (β1–β8) in blue. The four zinc-binding residues from the PS and the CD are shown for their side chains and labelled, as are the alanine-replacing catalytic Glu225, the first residue of mature mirolysin upon activation (Arg55), the Met-turn methionine (Met284) and Asp231, which replaces the canonical glycine of the zinc-binding motif. The catalytic zinc and the structural calcium cations are shown as magenta and blue spheres, respectively, the latter are labelled with their residue number, as are the N- and C-terminus. A green arrow pinpoints the final activation cleavage site (Ser54–Arg55). (*b*) Close-up in stereo of (*a*) after a horizontal 45° rotation downward depicting only segment Trp236–Tyr258, calcium ions Ca997 and Ca998 (blue spheres), and the liganding solvent molecules (red spheres). Calcium-coordinating atoms are connected by green lines, the solvents bridging the cations are linked with magenta lines. Residues involved in cation binding are labelled with their residue numbers. (*c*) Close-up in stereo of (*a*) after a 25° rotation to the left showing the active-site cleft of promirolysin, with the CD in cyan/blue (labels in blue) and the PS in pink/magenta (labels in magenta). PS segment Arg21–Asn28 is shown as a stick model for its main chain; selected side chains are further displayed (pink carbons), as are relevant side chains of the CD (pale blue carbons). The CD zinc ligands are not labelled for clarity [see (*a*)]. (*d*) Close-up in stereo of (*a*) after a horizontal 30° rotation downward and a 50° rotation to the right. A green arrow pinpoints the final activation cleavage site (Ser54–Arg55).

Promirolysin E225A has a compact structure of approximate dimensions 60 × 45 × 45 Å, and it splits into an N-terminal 34-residue PS (Arg21–Ser54) and a downstream 274-residue metzincin-type CD (Arg55–Leu328). The latter subdivides into an upper N-terminal sub-domain (NTSD; Arg55–Asp231 + Leu306–Leu328) and a lower C-terminal sub-domain (CTSD; Leu232–Ser305) separated by a horizontal active-site cleft (Fig. 2 ▸). The PS consists of two contiguous perpendicular extended segments, I (Arg21–Gly24) and II (Ser25–Asn28), followed by helices α1p (Met29–Thr35) and α2p (Pro37–Leu52), which are rotated by ∼60° relative to each other and connected by linker residue Glu36 (for secondary-structure nomenclature, see Fig. 1 ▸). After α2p, segment His53–Set54 leads to the primary activation site [Figs. 2 ▸(*a*), 2 ▸(*c*) and 2 ▸(*d*)]. In addition, the extended segment II and α1p are roughly perpendicular to each other because of a kink downward mediated by Gly24. The PS moiety is held together by a hydrophobic core made up of Leu27, Met29 and Ile32 from α1p; and Lys39, Tyr40 and Ile43 from α2p. In addition, the side chains of Arg33 from α1p and Tyr40 from α2p provide a stacking interaction at the protein surface.

The NTSD contains a strongly twisted and arched five-stranded β-sheet arranged top to bottom [Fig. 2 ▸(*a*)], in which the top strand is split into two (strands β2 and β3) by a protruding loop (Lβ2β3) called the LNR-like loop (Tallant *et al.*, 2006 ▸). The remaining strands (β1, β4, β8 and β7) are continuous and parallel to the top strand, except for the lowermost strand β7 which is antiparallel and forms the upper rim of the active-site cleft. Two roughly perpendicular helices, the backing-helix α1 and the active-site helix α4, nestle on the concave face of the sheet, and a third one, the second C-terminal helix α6, is attached to the convex face of the sheet near strands β1 and β2 [Fig. 2 ▸(*a*)]. Loop Lα1β2 extends down to the CTSD and includes two short helices, α2 and α3. The active-site helix contains the first residues of the zinc-binding motif of metzincins and includes His224 and His228, which coordinate the catalytic zinc (Zn999) at the bottom of the active-site cleft, as well as glutamate-replacing Ala225. The NTSD finishes at Asp231, which is normally a glycine in metzincins (Bode *et al.*, 1993 ▸; Cerdà-Costa & Gomis-Rüth, 2014 ▸), with a sharp turn downward and enters the CTSD. This sub-domain contains little regular secondary structure except for helices α2, α3 and the C-terminal helix α5, and it provides the third zinc ligand of the metzincin motif, His234. The three histidines bind the metal through their respective N^∊2^ atoms at distances spanning 1.95–2.07 Å in the four protomers, which are typical values for Zn–N bonds (2.03 Å on average) (Harding, 2006 ▸). Another characteristic element of metzincin CTSDs is the Met-turn (Asn282–Asp285), which forms a hydrophobic base for the zinc site (Tallant, García-Castellanos *et al.*, 2010 ▸). Moreover, atom O^η^ of the downstream residue Tyr286 is close to the zinc but too far apart for coordination (4.62 Å). Tyrosines in similar positions are zinc ligands in unbound members of the astacin and serralysin families of metzincins, a function that was also proposed for ulilysin. They are swung out upon substrate binding in a motion referred to as tyrosine switch (Baumann *et al.*, 1995 ▸; Tallant *et al.*, 2006 ▸; Gomis-Rüth, Trillo-Muyo *et al.*, 2012 ▸).

Structural cohesion of the CD is provided by two internal disulfides, Cys243–Cys271 and Cys262–Cys291, and a double structural calcium site, which further explains the calcium dependence of the enzyme (Koneru *et al.*, 2017 ▸). The two cations are liganded by residues from segment Trp236–Tyr258, which adopts a double S-loop structure, and by solvents [Fig. 2 ▸(*b*)]. Ca997 is bound in an octahedral plus one coordination by seven oxygens at distances spanning 2.28–2.49 Å, which are typical values for Ca–O bonds (2.36–2.39 Å on average) (Harding, 2006 ▸). Atoms Gly256 O, Trp236 O, Ser242 O and a solvent are roughly in a plane with the cation, while Gln255 O and Asp239 O^δ1^ plus O^δ2^ are in the apical positions. Ca998 is octacoordinated by Thr252 O^γ1^, Ile249 O and three solvents coplanar with the cation, and by Asp247 O^δ1^ above the plane and Thr252 O plus a solvent below the plane. Liganding distances range from 2.33 to 2.57 Å. Thus, Ca997 is more tightly bound than Ca998, probably because of fewer coordinating solvents. Overall, the two cations are 9.0 Å apart bridged by three solvents [Fig. 2 ▸(*b*)].

### 3.3. Mechanism of latency   

The PS traverses the active-site cleft of mirolysin in the opposite direction of the substrate [Figs. 2 ▸(*a*), 2 ▸(*c*) and 2 ▸(*d*)]. This is a mechanism previously described for other MP zymogens that prevents cleavage as the Michaelis complex required for catalysis cannot be formed [see Section 3.4 and Arolas *et al.* (2018 ▸)]. Analysis of the PS–CD interaction surface revealed an associated calculated solvation free-energy gain upon formation of the interface of −11.1 kcal mol^−1^ according to Krissinel & Henrick (2007 ▸) and an interface of 1151 Å^2^, which corresponds to a buried surface area of 2302 Å^2^. These values account for a strong interaction that is wider than average for buried surfaces of protein–protein complexes (1910 Å^2^) (Janin *et al.*, 2008 ▸) and remarkable given the small size of the PS. Indeed, 144 atoms from 44 residues of the CD and 105 atoms from 20 residues of the PS participate in the interface, which includes 17 hydrogen bonds, three salt bridges and one metallorganic bond [see Table 2 ▸ and Figs. 2 ▸(*c*) and 2 ▸(*d*)]. Participating segments are Arg55–Val57, Met147, Asp179–Thr192, Tyr216–Gly240, Ser255, Asn248, Tyr258–Glu269, Asp285–Met292, and segment Arg302–Ile313 from the CD; and Arg21–Glu30 plus Lys39–Ser54 from the PS.

A series of interactions are performed by Arg21, which belongs to the extended segment I and occupies the S_1_′ site of the cleft [Fig. 2 ▸(*c*)]. Its α-amino group hydrogen-bonds Tyr216 O^η^ and Tyr286 O, while its side chain fixes Thr221 O^γ^ plus Thr287 O, and salt-bridges Asp289 O^δ1^. This aspartate is key for substrate specificity [see Section 3.5 and Koneru *et al.* (2017 ▸)]. Tyrosine-switch Tyr286 pinches the extended segment I together with the upper-rim-strand segment Leu181–Tyr183 of the CD. In the capital interaction for latency, downstream Cys23 S^γ^ binds the catalytic zinc at 2.11–2.22 Å in the different structures, which is closer than typical Zn–S distances (2.31 Å) (Harding, 2006 ▸), and contributes together with the three histidine ligands to a tetrahedral zinc coordination sphere [Figs. 2 ▸(*c*) and 2 ▸(*d*)]. Downstream residue Ser25 from the extended segment II binds the upper-rim strand through its side chain. Its carbonyl contacts Met147 S^δ^, which is found in two conformations. Residue Glu26 tightly binds tyrosine-switch Tyr286 O^η^ through its O^∊1^ atom, thus fixing the swung-out conformation of the aromatic ring, with the main chain of Leu27 fixed by Asp238. The hydrophobic core of the PS (see Section 3.2) is expanded through CD residues Phe186, Phe188 and Arg233, which glue the PS to the CD through hydrophobic forces [Fig. 2 ▸(*c*)]. This hydrophobic core is delimited in the back by Arg302 and Glu47, which are engaged in a double salt bridge. The core is further extended to the left by Trp46, which is buried in a hydrophobic pocket created by the CD residues Pro187, Phe188, Leu306 and Ile313, as well as Ile50 from the PS [Fig. 2 ▸(*d*)]. In addition, Trp46 N^∊^ is fixed by the Asp231 side chain, which maintains the side chain of Arg302 in a competent conformation for Glu47 binding. This contribution to the PS–CD interface explains why an aspartate replaces the glycine normally found here in metzincins as part of the zinc-binding motif. Finally, the primary activation site Ser54–Arg55 is access­ible, and Arg55 protrudes from the molecular surface by virtue of a hydrogen bond between Arg55 N and Asp308 O^δ2^. This residue further fixes the downstream segment of the CD through a second hydrogen bond (Asp308 O^δ2^⋯Ser56 N).

Superposition of mature ulilysin onto the CD of promiro­lysin gave an r.m.s.d. of 0.98 Å for 250 common C^α^ atoms, which reflects close structural similarity that is consistent with the 50% sequence identity observed (Fig. 1 ▸). Furthermore, ulilysin contains a cysteine–glycine motif at the beginning of the zymogen sequence with the same number of PS residues, as well as many of the aforementioned structural features. Thus, the zymogenic structure and mechanism derived here for mirolysin are probably valid for ulilysin and other unicellular pappalysins (Fig. 1 ▸).

### 3.4. Promirolysin latency in the context of other MPs   

Zymogenicity in MPs was first structurally analysed in the 1990s for the funnelin metallocarboxypeptidases (Coll *et al.*, 1991 ▸; Gomis-Rüth *et al.*, 1995 ▸; Gomis-Rüth, 2008 ▸) and for mammalian MMPs (Becker *et al.*, 1995 ▸; Morgunova *et al.*, 1999 ▸; Tallant, Marrero *et al.*, 2010 ▸). MMPs are found, often in several copies, in animals, plants, fungi, archaea, bacteria and viruses (Marino-Puertas *et al.*, 2017 ▸), with 23 paralogs in humans. Mammalian MMP zymogens are inhibited by 70–90-residue PSs upstream of the CD through a cysteine within a conserved motif, PRCG*X*PD. This residue binds the catalytic zinc and is engaged in a cysteine-switch or velcro mechanism (Springman *et al.*, 1990 ▸; Van Wart & Birkedal-Hansen, 1990 ▸; Massova *et al.*, 1998 ▸; Rosenblum *et al.*, 2007 ▸; Tallant, Marrero *et al.*, 2010 ▸; Arolas *et al.*, 2018 ▸), which probably functions in a similar manner in MMPs from other animals, plants and fungi (Marino-Puertas *et al.*, 2017 ▸). Activation does not lead to substantial re­arrangement, *i.e.* the CD and the active site are already preformed in the zymogen and just shielded by the PS. Thus, the results herein indicate that latency for promirolysin and uni­cellular pappalysins probably operates based on a cysteine switch featuring a cysteine imbedded here in a conserved cysteine–glycine motif (Fig. 1 ▸).

**Figure 3:**
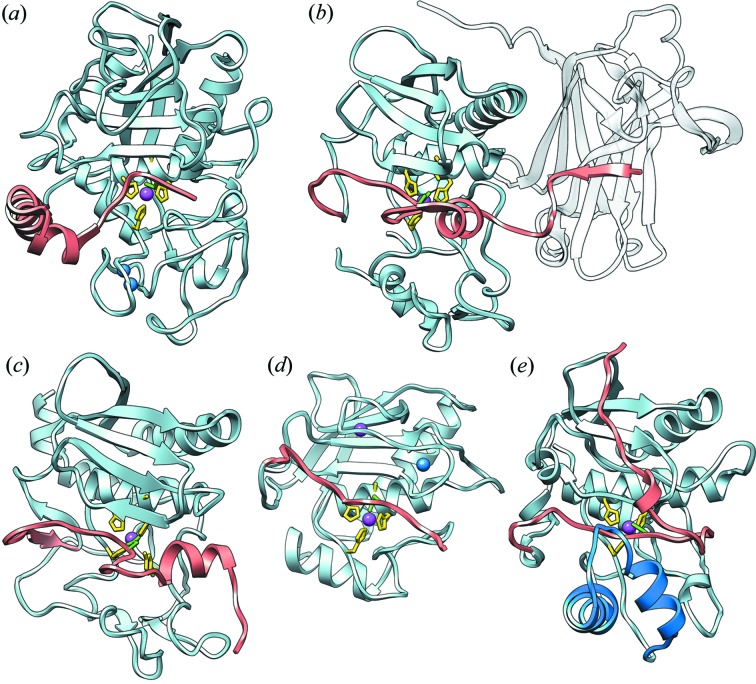
Metallopeptidase zymogens with short PSs. (*a*) A ribbon-type plot of *T. forsythia* promirolysin (PDB entry 6r7v; this work) with the PS in salmon and the CD in pale blue. The catalytic zinc and the structural calcium cations are shown as magenta and blue spheres, respectively. The side chains of the three histidine zinc ligands are shown as yellow sticks, the PS residue blocking the zinc is in green. (*b*) Same as (*a*) depicting human promeprin β (PDB entry 4gwm; Arolas *et al.*, 2012 ▸). The C-terminal TRAF domain, along which the N-terminal segment of the PS runs, is shown in white for reference. (*c*) *A. astacus* proastacin (PDB entry 3lq0; Guevara *et al.*, 2010 ▸). (*d*) *T. forsythia* prokarilysin (PDB entry 4r3v; López-Pelegrín *et al.*, 2015 ▸). (*e*) Promyroilysin from *Myroides* sp. CSLB8 (PDB entry 5gwd; Xu *et al.*, 2017 ▸). Uniquely among these MP zymogens, the PS is covered here by a flap (Thr160–Asp193, in blue), which is folded outward upon activation to liberate the cleft.

Latency in MPs through short N-terminal PSs (<50 residues), running in the opposite direction to the substrate across the active-site cleft as in promirolysin, occurs in other metzinc­ins (Fig. 3 ▸) (Arolas *et al.*, 2018 ▸). These include members of the astacin family, namely meprin β [Fig. 3 ▸(*b*)] from humans (PS of 44 residues, PS–CD interface 1225 Å^2^; Arolas *et al.*, 2012 ▸), archetypal astacin [Fig. 3 ▸(*c*)] from the crayfish *Astacus astacus* (34 residues, 1580 Å^2^; Guevara *et al.*, 2010 ▸), and myroilysin [Fig. 3 ▸(*e*)] from the bacterium *Myroides* sp. CSLB8 (37 residues, 1720 Å^2^; Xu *et al.*, 2017 ▸). Another example is the MMP karilysin [Fig. 3 ▸(*d*)] from *T. forsythia* (14 residues, 1050 Å^2^; Karim *et al.*, 2010 ▸; Cerdà-Costa *et al.*, 2011 ▸; Guevara *et al.*, 2013 ▸; López-Pelegrín *et al.*, 2015 ▸). In all cases, the PSs adopt mainly extended but markedly different conformations, even within families with closely related CDs, that cover large inter­action areas and run across the front surface of the CDs (Fig. 3 ▸). In pro­astacin and promeprin β, latency is exerted through an aspartate switch, with an aspartate from the PS blocking the zinc ion instead of a cysteine (Guevara *et al.*, 2010 ▸; Goulas *et al.*, 2011 ▸; Arolas *et al.*, 2012 ▸; Goulas & Gomis-Rüth, 2013 ▸). In contrast, promyroilysin has a cysteine switch [Fig. 3 ▸(*a*)]. Finally, in contrast to mammalian MMPs, the active site of the bacterial MMP karilysin is blocked by an aspartate switch. Thus, different mechanisms are found in the MMP and astacin families, with the zinc-blocking aspartates and cysteines presented into the active sites by disparate PS scaffolds [Figs. 3 ▸(*a*)–3 ▸(*e*)].

### 3.5. A mirolysin product complex   

**Figure 4:**
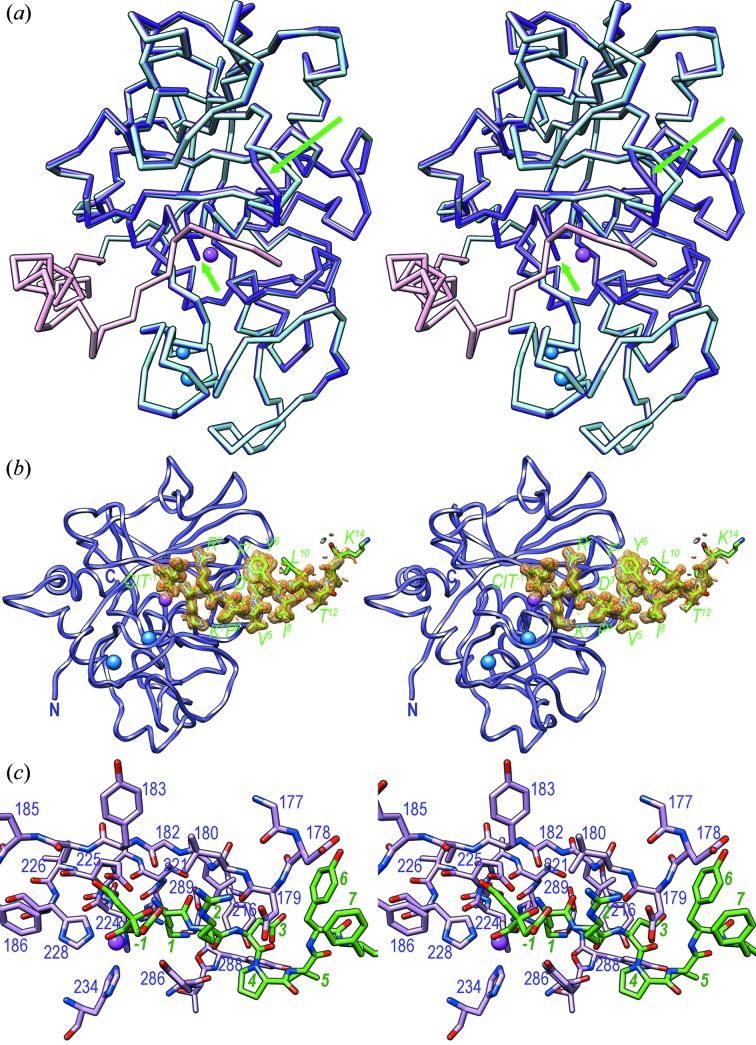
A product complex of mature mirolysin. (*a*) Superposition of the C^α^ plots of promirolysin (PS in pink, CD in light blue) and mature mirolysin (purple) in the orientation of Fig. 2 ▸(*a*). Significantly deviating regions are pinpointed by green arrows. The catalytic zinc and the structural calcium cations are shown as magenta and blue spheres, respectively. (*b*) Detail of the initial Fourier omit map to 1.5 Å of the product complex around the citrate (*CIT−1*) and the tetradecapeptide (*Lys1*–*Lys14*), both as stick models with green carbons and labels. The map (in orange) is contoured at 0.6σ above threshold and is clear for *CIT−1* and the main and side chains of *Lys1*–*Ile8* and *Ser11*–*Thr12*, as well as for the main chains of *Lys9*, *Leu10*, *Ile13* and *Lys14*. The view results from an ∼45° rotation downward from the standard orientation of Fig. 2 ▸(*a*). (*c*) Close-up view of mature mirolysin (carbons in plum) and the product (carbons in green) resulting from the view in (*a*) after a vertical 90° rotation to the left. Selected residues are labelled with their residue numbers in purple and dark green, respectively.

We next obtained a product complex of mature mirolysin with a tetradecapeptide (*Lys1*–*Lys14*) occupying S_1_′ and further primed sub-sites of the cleft plus a citrate in S_1_ (*CIT−1*). Superposition onto promirolysin revealed a core r.m.s.d. of 0.44 Å upon alignment of 269 out of the 270 protein residues of mature mirolysin and 307 residues of promirolysin [Figs. 4 ▸(*a*), 4 ▸(*b*) and 4 ▸(*c*)]. Thus, no major overall structural rearrangement occurred upon activation [Fig. 4 ▸(*a*)], as previously described for several MP families including MMPs and others (Tallant, Marrero *et al.*, 2010 ▸; López-Pelegrín *et al.*, 2015 ▸; Arolas *et al.*, 2018 ▸). A subtle rotation of the NTSD of ∼3° was detected, as well as rearrangement of the mature N-terminus, which protruded from the molecular surface and was disordered for its first three residues. In addition, segment Gly177–Asp179 underwent a downward motion (a maximal deviation of 2.45 Å at Asp178 C^α^) for substrate binding mediated by the flip of the peptide bond Leu176–Gly177. Upon removal of the PS, Asp231, which plays a key role in the zymogen (see Section 3.3), salt-bridged Arg233, whose side chain was rotated to meet the aspartate. Similarly, Arg302 was rearranged in the absence of its zymogenic salt-bridge partner Asp47 and contacted Asp247 (Arg302 N^η1^–Asp247 O, 3.08 Å). In addition, Thr311 was slightly lifted downwards because of the absence of the PS around His53.

Citrate *CIT−1* mimics an amino acid in S_1_ after catalysis. Its central quaternary carbon resembles the C^α^ atom. It is bound to a hydroxyl (*O7*), an α-carboxylate similar to that found after catalysis (with oxygen atoms *O5* and *O6*), a β-carboxylate as from an aspartate side chain (oxygens *O3* and *O4*) and a second β-carboxylate (oxygens *O1* and *O2*). The latter mimics substrate atoms upstream of the C^α^ in P_1_, and *O1* strongly binds general-base atom Glu225 O^∊1^ [Table 3 ▸ and Fig. 4 ▸(*c*)], indicating that either oxygen must be protonated, while *O2* contacts upper-rim atom Ala184 N. Atoms *O5* and *O6* bind the catalytic zinc in a distorted bidentate fashion. Moreover, *O5* weakly binds tyrosine-switch residue Tyr286 O^η^, thus suggesting a role for this residue in the stabilization of the tetrahedral reaction intermediate and/or product, as well as in zinc binding to the unbound enzyme. Finally, *O7* hydrogen-bonds the α-amino group of *Lys1* in sub-site S_1_′. This nitrogen further binds *CIT−1 O1*, general base Glu225 O^∊2^ and the upper-rim main-chain carbonyl of Gly182, but not the catalytic zinc [Fig. 4 ▸(*c*)]. The side chain of *Lys1* intrudes into the S_1_′ specificity pocket and binds the main-chain carbonyl of Thr287 at the pocket bottom. *Lys1 N^ζ^* is linked to Asp289 O^δ1^ through an internal solvent-mediated salt bridge, which explains the preference for basic residues in S_1_′ (Koneru *et al.*, 2017 ▸). An arginine, which contains two extra non-hydrogen side-chain atoms, would be directly bound by Asp289. The strong conservation of Asp289, which plays a major role in latency (see Section 3.3), across pappalysins [see Fig. 1 ▸ and Fig. 1 in the work by Tallant *et al.* (2006 ▸)] indicates that the specificity for basic residues in S_1_′ should be common for this family, as further shown for archaeal ulilysin (Tallant *et al.*, 2006 ▸) and human PAPP-A (Laursen *et al.*, 2001 ▸, 2002 ▸) and PAPP-A2 (Overgaard *et al.*, 2001 ▸). The carbonyl of *Lys1* binds the upper-rim main chain at Leu181. *Arg2* is in S_2_′ and thus points to bulk solvent. Its side chain is fixed by *CIT−1O3* and the side chain of Asp179, which is further engaged in binding the main-chain nitrogen of residue *Asp3* in S_3_′ through its main-chain carbonyl. The *Asp3* side chain contacts Tyr216 O^η^, and *Pro4* in S_4_′ weakly interacts with the Tyr258, Tyr286 and Glu260 side chains. Downstream *Val*5 is on the surface of the enzyme and the peptide chain turns upward so that the side chain of *Tyr6* in S_6_′ sticks to the molecular surface around Asp178–Asp179. From *Tyr6* onwards, the peptide adopts a helical structure until *Ile13*. From *Phe7* onwards, it does not interact with mirolysin [Fig. 4 ▸(*b*)]; instead, the peptide is fixed until *Lys14* by crystal contacts.

## 4. Conclusions   

Mirolysin, ulilysin and other unicellular pappalysins, which are present in archaea, bacteria, cyanobacteria, algae and fungi, most likely bind substrates in extended conformations from left (residues upstream of the scissile bond) to right (downstream residues) following the commonly accepted dogma (Madala *et al.*, 2010 ▸). The specificity of these and most MPs (Gomis-Rüth, Botelho *et al.*, 2012 ▸) is exerted by the S_1_′ specificity pocket, which in pappalysins accommodates substrate lysines and arginines because of the presence of a conserved aspartate at the bottom of the pocket.

Mirolysin and most likely other unicellular pappalysins utilize a zymogenic cysteine-switch mechanism exerted by a cysteine in a conserved cysteine–glycine dipeptide within the PS, which runs in the opposite direction to the substrate along the cleft, preventing cleavage and shielding the preformed competent CD. This is reminiscent of other metzincin families with short N-terminal PSs, *e.g.* the MMPs and astacins. In these families, aspartates may replace the cysteine in some but not all family members, and the conformations of the PSs vary largely. Overall, the results herein support the hypothesis that latency mechanisms are less conserved than the structure and mechanisms of the mature CDs.

Finally, the structural studies reported herein demonstrate substrate binding and zymogenicity for mirolysin, providing molecular mechanisms for biochemical reactions and latency of the pappalysin family of MPs within the metzincin clan. These data have practical implications in that PSs and bound substrates are templates for the design of specific and potent therapeutically active inhibitors (Lazure, 2002 ▸; Mittl & Grütter, 2006 ▸; Congreve *et al.*, 2005 ▸), *e.g.* against *T. forsythia*, a key player in PD.

## Supplementary Material

PDB reference: mature mirolysin product complex, 6r7w

PDB reference: selenomethionine-derivatized promirolysin, 6r7u

PDB reference: native promirolysin, 6r7v

## Figures and Tables

**Table 1:** Crystallographic data

	Promirolysin (SeMet)†	Promirolysin (SeMet)	Promirolysin (native)	Mirolysin (product complex)
Data processing				
Space group, protein molecules per asymmetric unit	*P*2_1_, 2	*P*2_1_, 2	*P*2_1_2_1_2_1_, 1	*P*2_1_2_1_2_1_, 1
Cell constants (*a*, *b* and *c* in Å, β in °)	47.78, 79.21, 75.50, 106.80	47.78, 79.21, 75.50, 106.80	47.33, 67.25, 79.64, 90.0	40.61, 66.49, 96.22, 90.0
Wavelength (Å)	1.2815	1.2815	1.2816	0.9792
No. of measurements/unique reflections	341819/134930	340267/70233	487729/50157	517385/41508
Resolution range (Å)‡	53.4–1.60 (1.70–1.60)	53.4–1.60 (1.70–1.60)	51.4–1.40 (1.48–1.40)	54.7–1.50 (1.59–1.50)
Completeness (%)	96.4 (96.0)	98.9 (99.1)	98.7 (92.0)	97.6 (90.4)
*R*_merge_	0.097 (0.857)	0.114 (0.983)	0.076 (1.041)	0.038 (0.235)
*R*_meas_/CC^1/2^	0.121 (1.068)/0.993 (0.635)	0.128 (1.104)/0.996 (0.709)	0.080 (1.119)/0.999 (0.719)	0.040 (0.245)/1.000 (0.989)
〈*I*/σ(*I*)〉 of unique reflections after merging	8.8 (1.7)	10.9 (2.4)	13.8 (1.8)	41.3 (14.8)
*B* factor (Wilson) (Å^2^)/average multiplicity	25.4/2.5 (2.4)	25.0/4.8 (4.7)	25.6/9.7 (7.1)	25.6/12.5 (11.7)
				
Structure refinement				
No. of reflections used in refinement (in test set)		69544 (688)	49412 (722)	40780 (728)
Crystallographic *R* factor/free *R* factor		0.186/0.220	0.161/0.188	0.144/0.158
Correlation coefficient *F*_obs_ − *F*_calc_ (test set)§		0.942 (0.915)	0.971 (0.960)	0.965 (0.967)
No. of protein residues/atoms/solvent molecules/non-covalent ligands		616/4856/445/2 Zn^2+^, 4 Ca^2+^, 4 glycerol, 1 boric acid	307/2430/274/1 Zn^2+^, 2 Ca^2+^, 4 glycerol	284/2239/348/1 Zn^2+^, 2 Ca^2+^, 2 ethanol, 1 citrate
R.m.s.d. from target values bonds (Å)/angles (°)		0.012/1.14	0.010/1.01	0.010/1.02
Average *B* factors (Å^2^) (overall/molecule A/molecule B)		21.7/20.0/22.1	25.4/24.3/—	17.9/15.2/32.2
All-atom contacts and geometry analysis¶				
Protein residues in favoured regions/outliers/all residues		602 (96.6%)/4††/623‡‡	304 (96.2%)/3††/316‡‡	284 (96.9%)/1††/293‡‡
Outlying rotamers/bonds/angles/chirality/planarity		12 (2.2%)/0/1/0/0	4 (1.4%)/0/0/0/0	3 (1.2%)/0/0/0/0
All-atom clashscore		2.8	1.9	0.4
PDB access code		6r7u	6r7v	6r7w

† For phasing, Friedel pairs were kept separately.

‡ Data processing values in parentheses are for the outermost resolution shell.

§ According to the final *BUSTER*/*TNT* refinement step.

¶ According to the wwPDB X-ray structure validation report.

†† All outliers were unambiguously resolved in the final Fourier maps.

‡‡ Including residues with atoms in two positions.

**Table 2:** Electrostatic interactions of promirolysin at the PS–mature enzyme interface The first residue/atom belongs to the PS, the second to the CD. The distances are from the native promirolysin structure (PDB entry 6r7v).

Salt bridges (Å)	
Arg21 N^η2^–Asp289 O^δ1^	2.81
Glu47 O^∊1^–Arg302 N^η2^	2.77
Glu47 O^∊2^–Arg302 N^η1^	2.86
	
Metallorganic interactions (Å)	
Cys23 S^γ^–Zn999	2.22
	
Hydrogen bonds (Å)	
Arg21 N⋯Tyr216 O^η^	3.11
Arg21 N⋯Tyr286 O	3.32
Arg21 N^∊^⋯Thr287 O	3.44
Arg21 N^η1^⋯Thr221 O^γ1^	3.02
Arg21 N^η2^⋯Thr287 O	2.95
Thr22 O^γ1^⋯Asp179 O	2.55
Thr22 O^γ1^⋯Leu181 N	2.90
Thr22 O⋯Gly182 N	3.98
Gly24 N⋯Gly182 O	3.05
Gly24 O⋯Met147 S^δ^	3.24
Ser25 O^γ^⋯Ala184 N	2.86
Ser25 O^γ^⋯Ala184 O	2.84
Glu26 O^∊1^⋯Tyr286 O^η^	2.73
Leu27 N⋯Asp238 O^δ2^	2.87
Asn28 N^δ2^⋯Asp238 O	3.25
Trp46 N^∊1^⋯Asp231 O^δ1^	3.46
Trp46 N^∊1^⋯Asp231 O^δ2^	2.92

**Table 3:** Electrostatic interactions of mirolysin at the product–CD interface The distances are from the mirolysin product complex structure (PDB 6r7w).

Salt bridges (Å)	
*CIT−1 O3*–*Arg2 N^∊^*	2.85
*CIT−1 O3*–*Arg2 N^η2^*	3.17
*CIT−1 O1*–*Lys1 N*	3.14
*Lys1 N*–Glu225 O^∊2^	2.70
*Arg2 N^η1^*–Asp179 O^δ1^	2.83
	
Metallorganic interactions (Å)	
*CIT−1 O5*–Zn999	1.93
*CIT−1 O6*–Zn999	2.63
	
Hydrogen bonds (Å)	
*CIT−1 O1*⋯Glu225 O^∊1^	2.46
*CIT−1 O2*⋯Ala184 N	2.78
*CIT−1 O5*⋯Tyr286 O^η^	3.45
*CIT−1 O7*⋯*Lys1 N*	2.74
*Lys1 N*⋯Gly182 O	2.79
*Lys1 O*⋯Leu181 N	2.86
*Asp3 N*⋯Asp179 O	2.84
*Asp3 O^δ1^*⋯Tyr216 O^η^	2.69
